# Navigating the Neurological Abyss: A Comprehensive Review of Organophosphate Poisoning Complications

**DOI:** 10.7759/cureus.54422

**Published:** 2024-02-18

**Authors:** Aniket Patel, Gajanan Chavan, Anmol K Nagpal

**Affiliations:** 1 Emergency Medicine, Jawaharlal Nehru Medical College, Datta Meghe Institute of Higher Education & Research, Wardha, IND

**Keywords:** public health, prevention, rehabilitation, antidotes, cholinergic crisis, organophosphate poisoning

## Abstract

Organophosphate poisoning is a significant global health concern with implications for both occupational and environmental settings. This comprehensive review thoroughly explores the biochemical basis, clinical presentation, diagnostic methods, treatment strategies, and long-term effects of organophosphate exposure. The acute phase is characterized by cholinergic crisis, respiratory distress, and neurological manifestations, while delayed complications include the intermediate syndrome and organophosphate-induced delayed neuropathy. Diagnostic approaches involve clinical evaluation, cholinesterase-level assessments, and imaging studies. Treatment strategies encompass decontamination, antidotes such as atropine and pralidoxime, and supportive care. Long-term effects may include cognitive and neurological sequelae, necessitating rehabilitation approaches such as physical and occupational therapy. Prevention strategies include stringent occupational safety guidelines, sustainable agricultural practices, and public awareness initiatives. The implications for clinical practice underscore the importance of a multidisciplinary approach. At the same time, the call to action emphasizes the need for collaborative efforts in prevention and awareness to mitigate the impact of organophosphate poisoning on public health and the environment.

## Introduction and background

Organophosphate poisoning is toxicity from exposure to organophosphate compounds commonly found in pesticides, herbicides, and specific industrial products. These compounds exert their toxic effects by inhibiting acetylcholinesterase, a crucial enzyme responsible for acetylcholine breakdown in the nervous system [1]. This inhibition results in acetylcholine accumulation, leading to overstimulation of cholinergic receptors and a spectrum of symptoms varying from mild to severe [1]. The poisoning can occur through ingestion, inhalation, or dermal contact, each with varying degrees of toxicity contingent on the specific organophosphate compound. A profound comprehension of the molecular mechanisms behind organophosphate poisoning is imperative for precise diagnosis and effective treatment strategies [2].

Sources of organophosphate exposure encompass occupational settings, intentional ingestion, accidental food contamination, and pesticide exposure in domestic and industrial contexts [2,3]. The incidence of global organophosphate-related human exposures appears underestimated, with reported mortality rates ranging from 3-25% worldwide [2,3]. There are approximately 8000 exposures annually in the United States, resulting in few deaths [2]. Familiar sources include insecticides (e.g., malathion, parathion), nerve gases (e.g., sarin, VX), ophthalmic agents (e.g., echothiophate, isoflurophate), antihelmintics (e.g., trichlorfon), herbicides (e.g., tribufos, merphos), industrial chemicals (e.g., tricresyl phosphate), and contaminated food sources (e.g., apples, celery, bell peppers) [2].

Symptom severity hinges on the ingested amount, absorption route, and metabolic breakdown rate of the insecticide [2]. Timely diagnosis and treatment are pivotal to mitigate poisoning severity and enhance patient outcomes [2,4]. Notably, while agricultural pesticides are the primary source of exposure, household items like ant and roach sprays also contain organophosphate compounds [2]. Organophosphate poisoning can result from diverse sources, and symptom severity is influenced by factors such as exposure type and healthcare accessibility. Diligence in identifying and managing exposure is crucial to prevent and mitigate complications associated with organophosphate poisoning [2].

This comprehensive review aims to synthesize existing knowledge on organophosphate poisoning complications, serving as a consolidated resource for healthcare professionals, researchers, and policymakers. This review seeks to contribute to a holistic understanding of organophosphate poisoning by exploring biochemical basis, clinical presentation, diagnostic methods, complications, treatment strategies, and long-term effects. Additionally, we will delve into current research trends and future directions, fostering awareness and enabling informed decision-making in clinical care and public health.

## Review

Biochemical basis of organophosphate poisoning

Mechanism of Action

Inhibition of acetylcholinesterase: At the heart of organophosphate toxicity lies the potent inhibition of acetylcholinesterase (AChE), a crucial enzyme in the cholinergic neurotransmission system [5]. Acetylcholinesterase normally hydrolyses acetylcholine, a neurotransmitter, into acetate and choline, thereby terminating the signal at cholinergic synapses. Organophosphates irreversibly bind to the active site of acetylcholinesterase, forming a covalent bond and impeding its enzymatic activity. The consequence of AChE inhibition is the accumulation of acetylcholine in the synaptic cleft, leading to sustained stimulation of cholinergic receptors. This overstimulation gives rise to a cascade of symptoms, ranging from mild, such as nausea and headaches, to severe manifestations, like respiratory failure and convulsions [5].

Accumulation of acetylcholine: The accumulation of acetylcholine occurs due to the impaired breakdown caused by AChE inhibition. Acetylcholine acts as a neurotransmitter in the central and peripheral nervous systems, transmitting signals across synapses [6]. Excessive acetylcholine, as observed in organophosphate poisoning, leads to hyperactivity of cholinergic receptors, particularly nicotinic and muscarinic receptors. The effects of acetylcholine accumulation are widespread, affecting various organs and systems. Muscarinic receptor stimulation results in bradycardia, bronchoconstriction, and excessive salivation, while nicotinic receptor activation contributes to neuromuscular symptoms, including muscle fasciculations and weakness [6].

Metabolism and Breakdown of Organophosphates in the Body

Phase I metabolism: Phase I metabolism represents the initial step in the biotransformation of various substances within the body. This process involves introducing functional groups to the parent compound through oxidation, hydrolysis, or reduction reactions. These chemical modifications are usually catalyzed by enzymes, such as cytochrome P450, located in the endoplasmic reticulum of liver cells and other tissues [7]. Phase I metabolism aims to make the parent compound more reactive and, in many cases, to create sites for subsequent conjugation reactions in Phase II metabolism. The resulting metabolites generated in Phase I are often more polar than the original compound, which enhances their water solubility. This increased polarity is crucial for eliminating these metabolites from the body [7].

Phase II metabolism: Phase II metabolism involves conjugation reactions, where the polar metabolites generated earlier undergo attachment of water-soluble groups. This step is essential for further increasing the water solubility of the metabolites, making them highly hydrophilic and facilitating their efficient elimination from the body. Conjugation reactions commonly involve the addition of large, water-soluble molecules, such as glucuronic acid, sulfate, or amino acids, to the Phase I metabolites. These conjugation reactions are typically catalyzed by specific enzymes in the cytoplasm of liver cells and other tissues. The resulting conjugated metabolites are then excreted through urine or bile. The overall purpose of Phase II metabolism is to enhance the elimination of potentially harmful or toxic substances, contributing to the detoxification processes in the body and maintaining overall metabolic homeostasis. The coordination between Phase I and Phase II metabolism ensures the effective transformation and elimination of a wide range of xenobiotics and endogenous compounds, playing a crucial role in maintaining the body's equilibrium and protecting it from the adverse effects of various substances [7].

Clinical presentation

Acute Symptoms

Cholinergic crisis: Cholinergic crisis is a hallmark manifestation of organophosphate poisoning arising from the disruption of acetylcholinesterase activity. This enzyme typically breaks down acetylcholine, but organophosphates inhibit its function, accumulating acetylcholine at nerve synapses. This excess acetylcholine triggers a cholinergic crisis characterized by a cascade of symptoms affecting multiple organ systems. Its prominent clinical features are Salivation, Lacrimation, Urination, Defecation, GI upset, and Emesis (SLUDGE). Patients may experience heightened salivation, excessive tearing, involuntary urination, and defecation, indicative of autonomic nervous system overstimulation. Additionally, muscle twitching, miosis (constricted pupils), and gastrointestinal cramps contribute to the complex clinical presentation [6].

Respiratory distress: Respiratory distress is a critical and potentially life-threatening aspect of acute organophosphate poisoning. Excessive acetylcholine stimulation in respiratory muscles leads to bronchoconstriction, increased bronchial secretions, and, in severe cases, paralysis of respiratory muscles. This results in dyspnea, wheezing, and chest tightness [8].

Neurological manifestations: Acute neurological manifestations in organophosphate poisoning stem from the overstimulation of both the central and peripheral nervous systems. Patients may present with confusion, heightened agitation, seizures, and, in severe instances, a state of coma. The neurological symptoms are diverse and often multifaceted, reflecting the widespread impact of organophosphates on neurotransmission. These manifestations underscore the urgency of early recognition and intervention to mitigate the neurological consequences of poisoning. The complexity of neurological symptoms necessitates a comprehensive approach to clinical management, including neurological assessments, supportive care, and, when required, anticonvulsant therapy [9].

Delayed Symptoms

Intermediate syndrome: The intermediate syndrome is a distinctive phase that emerges one to four days after organophosphate exposure, adding complexity to the clinical course. This phase is characterized by a specific pattern of weakness and paralysis, with notable impact on the neck, shoulders, and proximal limb muscles. Respiratory muscles, in particular, may be vulnerable, potentially leading to respiratory failure. The intermediate syndrome necessitates ongoing monitoring, as the progression of weakness can be unpredictable. Supportive care during this phase is crucial, focusing on respiratory support, physical therapy, and measures to address the evolving neuromuscular challenges. The recognition and management of the intermediate syndrome significantly improve patient outcomes and prevent complications associated with respiratory compromise [10].

Organophosphate-induced delayed neuropathy (OPIDN): OPIDN represents a severe yet rare neurological complication that manifests days to weeks after exposure to organophosphates. This condition primarily affects the peripheral nervous system, leading to sensory and motor deficits. The most commonly impacted nerves are the long axons, resulting in symptoms ranging from tingling and numbness to muscle weakness and, in severe cases, paralysis. OPIDN poses diagnostic challenges due to its delayed onset and varied clinical presentation. Management strategies focus on symptomatic relief, rehabilitation, and, in some cases, neuroprotective agents. The understanding of OPIDN underscores the importance of long-term follow-up and ongoing neurological assessments for individuals with a history of organophosphate exposure [11].

Diagnostic methods

Clinical Evaluation

Clinical evaluation is a pivotal component in the initial assessment of individuals suspected of organophosphate poisoning, forming the bedrock for timely and accurate diagnosis. The process involves a meticulous examination of the patient's medical history, encompassing details of potential exposure, symptomatology, and the timeline of onset. Interrogating the patient about occupation, hobbies, recent chemical exposures, and any history of intentional or accidental ingestion provides critical contextual information. An accurate timeline of symptom development aids in determining the phase of poisoning and guides subsequent diagnostic and treatment decisions [12]. The physical examination, an essential aspect of clinical evaluation, is tailored to identify classic signs associated with organophosphate poisoning. Special attention is given to manifestations of the acute cholinergic crisis, including excessive salivation, lacrimation, urination, defecation, GI upset, and emesis, as well as respiratory distress and neurological abnormalities. Monitoring vital signs, such as respiratory rate, heart rate, blood pressure, and oxygen saturation, plays a crucial role in gauging the severity of poisoning. Regular assessment of neurological status helps track the progression of symptoms. The comprehensive nature of clinical evaluation sets the stage for subsequent diagnostic investigations and the initiation of timely interventions, significantly influencing patient outcomes [12].

Laboratory Tests

Cholinesterase levels: Measurement of cholinesterase activity stands as a crucial diagnostic tool for confirming organophosphate poisoning. Cholinesterase, an enzyme responsible for the breakdown of acetylcholine in the nervous system, is a primary target of organophosphate inhibition. Blood tests are conducted to assess cholinesterase levels, focusing on acetylcholinesterase and butyrylcholinesterase. These tests provide quantitative data on the degree of enzyme inhibition, offering valuable insights into the extent of exposure and the severity of organophosphate poisoning. Reducing cholinesterase levels is a supportive indicator of organophosphate exposure and aids in determining the urgency and intensity of medical intervention required [3].

Blood and urine organophosphate levels: Direct measurement of organophosphate concentrations in blood and urine is an additional layer of confirmation for exposure. Analysis of these biological samples provides information on the presence and quantity of specific organophosphate compounds, contributing to the overall diagnostic precision. It is important to note, however, that the effectiveness of these tests may be influenced by factors such as the timing of sample collection about the exposure, the specific organophosphate compound involved, and individual variations in metabolism. While these tests offer valuable diagnostic information, their limitations should be considered, especially in cases of delayed presentation or when the exposure occurred sometime before testing. Despite these considerations, blood and urine organophosphate level measurements play a significant role in confirming exposure and guiding the medical response in cases of suspected organophosphate poisoning [13].

Imaging Studies

Brain imaging for neurological complications: When neurological symptoms persist or become prominent in cases of organophosphate poisoning, the application of brain imaging studies is a valuable adjunct to clinical assessment. Techniques such as computed tomography (CT) and magnetic resonance imaging (MRI) provide detailed insights into the structural aspects of the brain, detecting potential abnormalities and evaluating neurological complications. Using X-rays, CT scans effectively identify acute conditions such as hemorrhage, edema, or structural irregularities [14]. On the other hand, MRI, utilizing powerful magnets and radio waves, is adept at generating detailed images of soft brain tissues, enabling a nuanced assessment of subtle abnormalities. In the context of organophosphate poisoning, these imaging studies play a crucial role in ruling out alternative causes for neurological symptoms, guiding therapeutic strategies, and ensuring a comprehensive understanding of the impact of organophosphates on the central nervous system. The integration of CT and MRI contributes to timely and targeted interventions, ultimately enhancing the management and outcomes for individuals affected by organophosphate poisoning [14].

Complications of organophosphate poisoning

Respiratory Complications

Respiratory failure: Respiratory failure is a critical and potentially life-threatening complication that can arise in the context of organophosphate poisoning. The excessive stimulation of cholinergic receptors in respiratory muscles, caused by inhibiting acetylcholinesterase by organophosphates, can lead to bronchoconstriction and respiratory muscle paralysis. This progression may result in inadequate ventilation, compromised gas exchange, and respiratory failure. Timely and appropriate respiratory support is crucial in managing this complication. Mechanical ventilation, which assists or replaces spontaneous breathing, becomes essential to maintain adequate oxygenation and prevent hypoxia. Close monitoring of respiratory parameters and prompt intervention is imperative to address respiratory failure promptly and optimize patient outcomes [15].

Pneumonia: Pneumonia represents a secondary complication that may arise in the aftermath of respiratory compromise in organophosphate poisoning. The inability to effectively clear airway secretions due to muscle weakness and impaired cough reflex increases the risk of aspiration and subsequent pneumonia [16]. Furthermore, the neurotoxic effects of organophosphates may contribute to impaired host defense mechanisms, further predisposing individuals to respiratory infections. Vigilant monitoring for signs of pneumonia, such as fever, cough, and abnormal chest X-ray findings, is essential. Early intervention, including administering appropriate antibiotics and respiratory support, is crucial to prevent and manage pneumonia effectively. Comprehensive care involves addressing the immediate consequences of respiratory failure and mitigating potential complications that can arise, emphasizing the importance of a multidisciplinary approach in managing organophosphate poisoning [16].

Cardiovascular Complications

Bradycardia: Cardiovascular complications in organophosphate poisoning often present as bradycardia, characterized by an abnormally slow heart rate. Excessive cholinergic stimulation, resulting from inhibiting acetylcholinesterase by organophosphates, leads to an uncontrolled increase in acetylcholine levels. This heightened cholinergic activity negatively impacts the sinoatrial node, the heart's natural pacemaker, causing a decrease in heart rate. In severe cases, bradycardia can contribute to hemodynamic instability and compromise cardiac output. Careful cardiac monitoring is essential in identifying and addressing bradycardia promptly. Atropine administration is a critical intervention in managing organophosphate poisoning, as it competitively blocks acetylcholine receptors, counteracting the cholinergic effects and restoring a more regular heart rate [17].

Hypotension: Hypotension, or low blood pressure, is another cardiovascular complication of organophosphate poisoning. The vasodilatory effects of accumulated acetylcholine and adverse inotropic effects on the heart contribute to a drop in blood pressure. The systemic vasodilation further exacerbates the hemodynamic instability observed in affected individuals. Adequate fluid resuscitation is crucial to managing hypotension, aiming to optimize intravascular volume and improve perfusion. In some cases, vasopressor support may be required to maintain hemodynamic stability, mainly when fluid resuscitation alone is insufficient. The collaborative efforts of healthcare professionals, including toxicologists, emergency physicians, and intensivists, are essential in navigating the complex cardiovascular consequences of organophosphate poisoning [18].

Neurological Complications

Seizures: The overstimulation of the central nervous system in organophosphate poisoning can precipitate seizures, constituting a severe neurological complication. The excessive accumulation of acetylcholine at nerve synapses, due to the inhibition of acetylcholinesterase by organophosphates, disrupts normal neurotransmission and can trigger epileptic activity. Seizures further exacerbate respiratory distress, potentially leading to respiratory failure, and complicate the overall clinical course. Prompt administration of anticonvulsant medications, such as benzodiazepines or other appropriate agents, is essential in managing seizures associated with organophosphate poisoning. Additionally, supportive measures, including airway management and respiratory support, are crucial in mitigating the consequences of seizures and preventing further complications [19].

Neuropathy: Organophosphate-induced neuropathy is a delayed neurological complication that can manifest in the aftermath of exposure. This condition primarily affects the peripheral nervous system, leading to sensory and motor deficits. Patients may experience weakness, numbness, and, in severe cases, paralysis. Rehabilitation becomes a key component in managing organophosphate-induced neuropathy, focusing on physical therapy to improve muscle strength, coordination, and overall mobility. Ongoing neurological monitoring is essential to assess the progression of neuropathy and tailor rehabilitation strategies accordingly. The multifaceted nature of neuropathic symptoms underscores the importance of a comprehensive and multidisciplinary approach involving neurologists, rehabilitation specialists, and other healthcare professionals [20].

Gastrointestinal Complications

Abdominal pain: Abdominal pain is a prevalent gastrointestinal manifestation observed in organophosphate poisoning. This symptom arises from the overstimulation of smooth muscles in the gastrointestinal tract due to the excessive accumulation of acetylcholine. The resulting spastic contractions and increased peristalsis contribute to abdominal discomfort and pain. Managing abdominal pain is a crucial component of supportive care in organophosphate poisoning. This involves pain management strategies, such as analgesic medications, and addressing associated symptoms to enhance the patient's overall well-being [21].

Nausea and vomiting: Excessive cholinergic stimulation in organophosphate poisoning extends to the gastrointestinal system, leading to symptoms of nausea and vomiting. These manifestations further contribute to the clinical complexity of the condition. Nausea and vomiting can result in fluid and electrolyte imbalances, adding to the overall challenges in managing organophosphate poisoning. Supportive measures are essential in addressing these gastrointestinal complications. Antiemetic medications may be administered to alleviate nausea and vomiting, and fluid resuscitation becomes crucial to maintain hydration and correct any electrolyte disturbances. The combined approach of managing abdominal pain, nausea, and vomiting contributes to the overall supportive care strategy, aiming to alleviate symptoms and improve the patient's comfort during organophosphate poisoning [22].

Treatment strategies

Decontamination

Dermal decontamination: Dermal decontamination is a critical first step in managing organophosphate exposure. Removing contaminated clothing and thoroughly washing the skin help minimize dermal absorption of the toxic substance. Quick initiation of decontamination is crucial, as it prevents the continued absorption of organophosphates through the skin. This rapid intervention is essential to mitigate the potential systemic effects and reduce the overall toxicity of the exposure. Proper decontamination protocols, including protective equipment for responders, are pivotal in preventing further absorption and ensuring the safety of individuals exposed to organophosphates [12].

Ocular decontamination: Ocular organophosphate exposure requires immediate and thorough decontamination. Irrigation with copious amounts of water is essential to flush the toxic substance from the eyes. This not only helps prevent ocular complications but also contributes to reducing systemic absorption through the highly vascularised ocular tissues. Rapid and effective ocular decontamination is critical in preserving vision and minimizing the extent of ocular damage. Emergency eyewash stations and protocols for prompt irrigation are essential components of workplace safety measures, particularly in industries where organophosphates are used [23].

Gastric lavage and activated charcoal: In cases of organophosphate ingestion, gastric lavage may be considered a decontamination measure, mainly if performed shortly after exposure. This involves introducing and removing a solution into the stomach to flush out the toxic substance. Additionally, activated charcoal may be administered to limit the absorption of organophosphates from the gastrointestinal tract. Activated charcoal acts as an adsorbent, binding the toxic substance and preventing its further absorption into the bloodstream. It's important to note that the effectiveness of these measures diminishes with time elapsed since ingestion. Therefore, prompt initiation of decontamination procedures is crucial for maximizing their efficacy in reducing systemic toxicity [24].

Antidotes

Atropine: Atropine is a pivotal antidote in managing organophosphate poisoning. Its mechanism of action involves the competitive blockade of acetylcholine receptors, effectively counteracting the excessive stimulation caused by the inhibition of acetylcholinesterase by organophosphates. Atropine is particularly effective in treating manifestations such as bradycardia, bronchoconstriction, and excessive secretions resulting from cholinergic overstimulation. Dosing is titrated based on the severity of symptoms, with careful monitoring for adverse effects. By antagonizing the effects of acetylcholine, atropine plays a crucial role in restoring physiological balance and mitigating the potentially life-threatening consequences of organophosphate toxicity [25].

Pralidoxime: Pralidoxime (2-PAM), is another essential antidote in managing organophosphate poisoning. Its primary mechanism of action involves reactivating acetylcholinesterase, which organophosphates have inhibited. By restoring enzymatic activity, pralidoxime facilitates the breakdown of accumulated acetylcholine, addressing muscarinic and nicotinic effects. Pralidoxime is most effective when administered early in the course of poisoning, and its use is often complemented by atropine. The combination of atropine and pralidoxime provides a comprehensive approach to counteract the diverse physiological impacts of organophosphates on the nervous system. Timing is critical for pralidoxime administration, emphasizing the importance of early recognition and intervention in managing organophosphate poisoning [26].

Supportive Care

Mechanical ventilation: In severe cases of organophosphate poisoning, respiratory failure may ensue due to respiratory muscle paralysis or bronchoconstriction. Mechanical ventilation becomes an indispensable intervention to ensure adequate oxygenation and ventilation. This life-saving measure supports respiratory function when the standard neuromuscular control of breathing is compromised. Close monitoring of respiratory parameters, such as oxygen saturation and end-tidal carbon dioxide, is essential. Timely intervention and adjustments in ventilator settings are crucial in preventing complications associated with respiratory distress, such as hypoxia and respiratory acidosis. Implementing mechanical ventilation requires a multidisciplinary approach involving critical care physicians, respiratory therapists, and nursing staff to optimize patient outcomes [19].

Fluid and electrolyte management: Organophosphate poisoning can disrupt fluid and electrolyte balance, primarily through excessive salivation, vomiting, and diarrhea. Supportive care includes fluid resuscitation to maintain adequate hydration and electrolyte balance. Intravenous fluids may be administered to replace losses and address potential dehydration. Electrolyte levels, especially potassium, should be closely monitored as organophosphates can impact ion channels and lead to imbalances. Prompt correction of electrolyte abnormalities is essential to prevent complications such as cardiac arrhythmias and neuromuscular dysfunction. Coordinating fluid and electrolyte management is integral to comprehensive care, requiring vigilant monitoring and adjustments based on the patient's clinical status [27].

Long-term effects and rehabilitation

Cognitive and Neurological Sequelae

Cognitive impairments: Cognitive impairments are common sequelae of organophosphate poisoning, affecting aspects of memory, attention, and executive functions. Patients may struggle with recalling information, sustaining attention on tasks, and engaging in complex cognitive processes. These impairments can significantly impact daily activities, work performance, and overall quality of life. Targeted interventions, such as cognitive rehabilitation and neuropsychological therapy, may be necessary to address specific cognitive deficits. Comprehensive assessments by neurologists or neuropsychologists are instrumental in identifying the nature and extent of cognitive impairments, guiding the development of tailored interventions to support cognitive functioning [28].

Neuropathy: Chronic neuropathic symptoms often persist in individuals who have experienced organophosphate poisoning. These symptoms may include tingling, numbness, and muscle weakness, reflecting the long-term impact of organophosphate-induced delayed neuropathy (OPIDN). OPIDN damages the peripheral nervous system, affecting sensory and motor functions. Rehabilitation approaches like physical and occupational therapy are crucial in managing neuropathic symptoms and improving overall functional outcomes. Long-term monitoring and interdisciplinary collaboration between neurologists and rehabilitation specialists are essential to address the evolving nature of neuropathic symptoms [20].

Psychological effects: Organophosphate poisoning can have psychological repercussions, with some individuals developing anxiety, depression, or post-traumatic stress disorder (PTSD). The severity of psychological effects may be influenced by the degree of exposure, the perceived threat during the incident, and individual vulnerability factors. Psychiatric interventions, including counseling and pharmacotherapy, may be necessary to address mental health symptoms. Support from mental health professionals and family and community resources can aid in psychological recovery. Acknowledging and addressing psychological effects is integral to the holistic care of individuals affected by organophosphate poisoning, promoting resilience and facilitating a comprehensive recovery [29].

Rehabilitation Approaches

Physical therapy: Physical therapy is a crucial component of rehabilitation for individuals experiencing motor deficits and muscle weakness following organophosphate poisoning. Therapists employ a range of therapeutic exercises and interventions to enhance muscle strength, improve coordination, and promote overall mobility. The goal is to restore optimal physical function, enabling individuals to regain independence in their daily activities. Physical therapists work closely with patients to develop personalized exercise regimens that address specific motor challenges and facilitate the recovery of motor skills [30].

Occupational therapy: Occupational therapy plays a vital role in supporting individuals affected by organophosphate poisoning in regaining independence in daily activities. Occupational therapists collaborate with patients to address challenges related to cognitive impairments, fine motor skills, and activities of daily living (ADLs). Through targeted interventions, individuals learn adaptive strategies and engage in activities that enhance their ability to perform daily tasks, fostering improved functionality and quality of life [31].

Speech and language therapy: Speech and language therapy is beneficial for individuals who experience communication or swallowing difficulties due to organophosphate poisoning. Therapists work on exercises to improve speech clarity, language skills, and swallowing function. This specialized therapy addresses expressive and receptive communication challenges, promoting effective communication and addressing potential complications related to impaired swallowing [32].

Cognitive rehabilitation: Cognitive rehabilitation programs are designed to support individuals with cognitive impairments resulting from organophosphate poisoning. These programs focus on improving memory, attention, and executive functions through structured exercises and cognitive strategies. Cognitive rehabilitation is tailored to each individual's needs and deficits to enhance cognitive abilities and foster adaptive coping mechanisms. The goal is to optimize cognitive functioning, ultimately promoting greater independence and improved quality of life [33].

Quality of Life After Organophosphate Poisoning

Psychological counseling: Psychological counseling plays a pivotal role in supporting individuals affected by the psychological impact of organophosphate poisoning. Counselors and mental health professionals provide therapeutic interventions to help individuals cope with anxiety, depression, or post-traumatic stress disorder (PTSD) that may arise from the traumatic experience of poisoning. Through counseling sessions, individuals can explore and address the emotional challenges associated with organophosphate exposure, develop coping strategies, and work toward psychological recovery. This aspect of care contributes to the individual's holistic well-being, recognizing and addressing the interconnectedness of physical and mental health [34].

Community and social support: Building a robust support network within the community and among friends and family is crucial for individuals recovering from organophosphate poisoning. Social interactions and support play a significant role in promoting mental and emotional well-being. Connecting with others who may have experienced similar challenges can provide a sense of understanding and solidarity. Community and social support contribute to resilience, fostering a supportive environment that aids recovery and helps individuals reintegrate into their daily lives [35].

Long-term medical follow-up: Regular and long-term medical follow-up is essential for individuals who have experienced organophosphate poisoning. This involves ongoing assessments by neurologists, rehabilitation specialists, and other relevant healthcare professionals to monitor and manage persistent symptoms. These follow-up appointments allow for the tracking of neurological recovery, adjustment of rehabilitation plans, and the identification of any emerging health concerns. The continuity of care ensures that individuals receive appropriate medical attention, interventions, and support as needed throughout their recovery journey [36].

Prevention and public health measures

Occupational Safety Guidelines

Personal protective equipment (PPE): Implementing robust PPE protocols is crucial in industries where organophosphates are used. This includes consistently using protective clothing, gloves, masks, and goggles to minimize dermal and respiratory exposure. Proper selection, maintenance, and disposal of PPE are integral components of occupational safety measures, providing a physical barrier between workers and potential sources of organophosphate exposure [37].

Training and education: Providing comprehensive training to workers is essential in creating awareness of organophosphate hazards. Training programs should cover proper handling techniques, storage procedures, and emergency response protocols. Educating workers about the potential risks equips them to take proactive measures, follow safety guidelines, and respond effectively during accidental exposure. Continuous education and regular updates ensure that workers remain informed about the latest safety practices and preventive measures [38].

Engineering controls: Implementing engineering controls is a proactive approach to minimizing the risk of accidental exposure in occupational settings. This involves designing and installing enclosed systems, ventilation mechanisms, and automated processes to contain and control the release of organophosphate compounds. By engineering workspaces to reduce the dispersion of hazardous substances, organizations can create a safer environment for workers, minimizing the potential for acute and chronic exposure [39].

Regular monitoring and surveillance: Establishing routine monitoring and surveillance programs in occupational settings is essential for detecting potential contamination and exposure risks. Regular assessments of air quality, water sources, and soil can identify elevated levels of organophosphates, signaling potential hazards. Continuous monitoring allows for early identification of issues, enabling timely intervention to mitigate risks and protect the health and safety of workers. Surveillance programs are integral to a proactive occupational health and safety strategy [40].

Agricultural Practices and Regulations

Integrated pest management (IPM): Encouraging the adoption of IPM practices represents a sustainable and environmentally friendly approach to pest control. IPM emphasizes the targeted and judicious use of pesticides, reducing reliance on chemical agents, and incorporating biological controls. By integrating various pest management strategies, including cultural, biological, and mechanical controls, IPM seeks to optimize crop protection while minimizing environmental impact and the need for organophosphate-based pesticides [41].

Regulatory oversight: Governments and regulatory bodies play a crucial role in safeguarding public health and the environment by establishing and enforcing safety standards for using organophosphates in agriculture. This includes rigorously evaluating pesticide formulations, setting permissible exposure limits, and regularly updating safety regulations based on evolving scientific knowledge. Regulatory oversight ensures that the use of organophosphates aligns with the latest safety standards and that potential risks are continuously assessed and addressed [42].

Alternative pest control methods: Promoting the research and development of alternative pest control methods represents a proactive strategy to reduce reliance on organophosphates. This includes the exploration of biopesticides, which utilize natural enemies of pests, as well as the implementation of sustainable agricultural practices like crop rotation. Diversifying pest management strategies helps minimize the environmental impact of pesticide use and contributes to the preservation of ecosystem health [43].

Educational programs for farmers: Providing education and training programs for farmers is instrumental in enhancing awareness of safe pesticide use, storage, and disposal practices. Knowledgeable farmers are more likely to adopt practices that reduce the risk of organophosphate exposure. Educational initiatives may cover proper application techniques, selecting less toxic alternatives, and adhering to recommended safety measures. Empowering farmers with information contributes to responsible pesticide use and fosters a culture of environmental stewardship in agriculture [44].

Public Awareness and Education

Community outreach programs: Engaging communities through outreach programs is crucial for raising awareness about the potential risks associated with organophosphates. These programs can include educational workshops, community meetings, and distribution of informational materials. The focus is educating residents near agricultural areas about safe practices, recognizing early signs of exposure, and responding appropriately to potential incidents. Community involvement is critical to building a shared understanding of the risks and fostering a collective commitment to safety [45].

Labeling and information campaigns: Clear and comprehensive labeling of products containing organophosphates and information campaigns are essential for empowering consumers to make informed decisions. Product labels should guide the proper storage, use, and disposal of organophosphate-containing products. Information campaigns can utilize various media channels to disseminate essential safety messages, reaching a broad audience and reinforcing the importance of following recommended practices for handling these substances [46].

School education programs: Incorporating information about pesticide safety, including organophosphates, into school curricula ensures students know the potential hazards and preventive measures. Educational programs can cover topics such as the safe use of pesticides in agriculture, recognizing warning signs of exposure, and understanding the importance of environmental stewardship. By integrating this information into school education, future generations are equipped with the knowledge to make informed choices about their environment and contribute to promoting safer practices [47].

Public health campaigns: Collaborative efforts between public health agencies, non-governmental organizations, and media outlets can contribute to widespread awareness through public health campaigns. These campaigns may use various channels, such as social media, posters, and community events, to disseminate information about organophosphate risks. Emphasizing the importance of adopting preventive measures, recognizing symptoms of exposure, and seeking timely medical attention can empower the public to prioritize safety and well-being [48].

Current research and future directions

Ongoing Studies on Organophosphate Poisoning

Several studies have delved into various aspects of organophosphate poisoning, each contributing valuable insights from different regions. In South India, a study aimed to unravel the sociodemographic characteristics of organophosphate-poisoned patients, investigating the source, site, and route of poisoning, alongside assessing serotonin and dopamine levels in plasma samples [49]. Meanwhile, in Turkey, a retrospective study scrutinized hospital records of patients admitted to an intensive care unit following acute organophosphate poisonings. This study focused on demographics, severity, complications, and overall outcomes [50]. Similarly, a cross-sectional study in South Africa explored the epidemiology of organophosphate poisoning in the Tshwane District, analyzing demographics, circumstances of poisoning, and case outcomes [51]. In Karachi, Pakistan, a study conducted at the National Poisoning Control Centre evaluated the demographics, severity scores, and outcomes of organophosphate poisoning cases, further contributing to the comprehensive understanding of this toxicological concern [52].

Emerging Therapies and Interventions

Research efforts in the field of organophosphate poisoning focus on several key areas. Firstly, there is a concerted effort towards developing novel antidotes or therapeutic interventions to counter the adverse effects of organophosphates on the nervous system. This involves exploring innovative strategies to mitigate the impact of these toxic compounds on neural function. Additionally, alternative treatments, including the use of atropine and other anticholinergic agents, are being investigated as potential approaches to manage the symptoms arising from organophosphate poisoning effectively. These alternatives aim to provide diversified and targeted therapeutic options for individuals affected by exposure to these toxic substances. Furthermore, ongoing research delves into the identification and study of potential neuroprotective agents, seeking to minimize the severity of neurological complications associated with organophosphate poisoning. Exploring neuroprotective interventions represents a proactive approach toward enhancing the overall outcomes and quality of care for individuals exposed to organophosphates [3].

Areas for Further Research

In the realm of organophosphate poisoning, several avenues of research are being explored to deepen our understanding and enhance outcomes. Firstly, there is a call for further investigation into the socio-demographic factors influencing the incidence and outcomes of organophosphate poisoning across diverse populations and regions. This research aims to uncover potential variations and nuances in susceptibility, response, and recovery, contributing to more targeted preventive and therapeutic approaches tailored to specific contexts [8]. Secondly, a critical focus lies in examining the effectiveness of existing treatments and interventions for organophosphate poisoning. Concurrently, there is an emphasis on developing novel therapeutic strategies to address gaps in current approaches. This dual-pronged research direction seeks to optimize the management of organophosphate poisoning, ensuring that interventions are not only efficacious but also adaptable to varying scenarios and patient profiles [53].

Additionally, researchers are delving into the intricate role of neurotransmitters, such as serotonin and dopamine, in the pathophysiology of organophosphate poisoning. This exploration extends to evaluating these neurotransmitters' potential utility as biomarkers for diagnosis and prognosis. Such investigations hold promise for refining diagnostic tools and prognostic indicators, facilitating more accurate and timely assessments of organophosphate poisoning cases [49]. Lastly, a crucial area of research involves assessing the broader impact of organophosphate poisoning on mental health and cognitive function. This includes the development of targeted interventions aimed at minimizing the complications that may arise in these domains. Understanding the psychological and cognitive sequelae of organophosphate exposure is pivotal in offering comprehensive care, addressing not only the immediate toxic effects but also the long-term well-being of affected individuals. Through these multifaceted research endeavors, the scientific community strives to advance knowledge, refine treatments, and improve outcomes for organophosphate poisoning patients [29].

## Conclusions

In conclusion, this comprehensive review has delved into the intricate dimensions of organophosphate poisoning, offering a nuanced understanding of its biochemical basis, clinical manifestations, diagnostic methods, treatment strategies, and long-term implications. Key findings underscore the urgency of recognizing acute symptoms such as cholinergic crisis and respiratory distress, as well as the potential for delayed complications like the intermediate syndrome and organophosphate-induced delayed neuropathy. Early and multidisciplinary interventions are pivotal in clinical practice for improved patient outcomes, emphasizing the importance of toxicologists, emergency physicians, neurologists, and rehabilitation specialists. On a broader scale, the implications for public health call for stringent occupational safety measures, sustainable agricultural practices, and robust regulatory oversight. The call to action involves empowering communities through education, advocating for regulatory measures, and fostering research and innovation in pest control. By collectively embracing these preventive and awareness measures, we can work towards a future where organophosphate poisoning is mitigated, safeguarding human health and environmental well-being.
